# Activated Human CD4+CD45RO+ Memory T-Cells Indirectly Inhibit NLRP3 Inflammasome Activation through Downregulation of P2X7R Signalling

**DOI:** 10.1371/journal.pone.0039576

**Published:** 2012-06-29

**Authors:** Vanessa Beynon, Francisco J. Quintana, Howard L. Weiner

**Affiliations:** Center for Neurologic Diseases, Brigham and Women’s Hospital, Harvard Medical School, Boston, Massachusetts, United States of America; University Paris Sud, France

## Abstract

Inflammasomes are multi-protein complexes that control the production of pro-inflammatory cytokines such as IL-1β. Inflammasomes play an important role in the control of immunity to tumors and infections, and also in autoimmune diseases, but the mechanisms controlling the activation of human inflammasomes are largely unknown. We found that human activated CD4+CD45RO+ memory T-cells specifically suppress P2X7R-mediated NLRP3 inflammasome activation, without affecting P2X7R-independent NLRP3 or NLRP1 inflammasome activation. The concomitant increase in pro-IL-1β production induced by activated memory T-cells concealed this effect. Priming with IFNβ decreased pro-IL-1β production in addition to NLRP3 inflammasome inhibition and thus unmasked the inhibitory effect on NLRP3 inflammasome activation. IFNβ suppresses NLRP3 inflammasome activation through an indirect mechanism involving decreased P2X7R signaling. The inhibition of pro-IL-1β production and suppression of NLRP3 inflammasome activation by IFNβ-primed human CD4+CD45RO+ memory T-cells is partly mediated by soluble FasL and is associated with down-regulated *P2X7R* mRNA expression and reduced response to ATP in monocytes. CD4+CD45RO+ memory T-cells from multiple sclerosis (MS) patients showed a reduced ability to suppress NLRP3 inflammasome activation, however their suppressive ability was recovered following *in vivo* treatment with IFNβ. Thus, our data demonstrate that human P2X7R-mediated NLRP3 inflammasome activation is regulated by activated CD4+CD45RO+ memory T cells, and provide new information on the mechanisms mediating the therapeutic effects of IFNβ in MS.

## Introduction

IL-1β is a potent cytokine that acts on different cell types to induce a proinflammatory response [1], thus the production of active IL-1β is tightly regulated. Familial autoinflammatory syndromes, such as Muckle-Wells-Syndrome, are linked to excessive secretion of IL-1β and have helped to elucidate the mechanisms that regulate the secretion of active IL-1β [2]. The secretion of active IL-1β is controlled by a sophisticated multistep process [3], [4] in which the *IL1B* promoter is first transactivated in response to different stimuli such as toll-like receptor (TLR) ligands. In a second step, multiprotein complexes, termed inflammasomes, are assembled and catalyze the maturation of IL-1β. Nucleotide oligomerization domain receptors (NLRs) are central components in the majority of inflammasomes, which are complexed with other proteins to form active inflammasomes in response to a plethora of exogenous and endogenous ligands such as ATP, alum or monosodium urate (MSU) crystals [5]. Once activated, the inflammasomes catalyze the proteolytic maturation of caspase-1, which then cleaves pro-IL-1β to IL-1β [3], [6].

IL-1β is important for the differentiation and survival of Th17 cells [7], [8], [9], [10]. The important role played by Th17 cells in the pathogenesis of multiple sclerosis (MS) suggests that inflammasome activation contributes to the pathogenesis of the disease. Indeed, the generation of active IL-1β by caspase-1 controls the development of experimental autoimmune encephalomyelitis (EAE), an experimental model of MS [11]. Furthermore, elevated levels of caspase-1 expression are found in MS plaques and in the peripheral blood mononuclear cells (PBMCs) of MS patients [12], [13]. Although the control of inflammasome activation plays an important role in the generation of active IL-1β and the encephalitogenic immune response, the mechanisms that regulate the activity of human inflammasomes are largely unknown.

Interferon-β (IFNβ) is a first line therapy in the treatment of relapsing-remitting multiple sclerosis (MS) [14], [15], [16]. Early intervention with IFNβ decreases the frequency and severity of relapses, reduces the number of brain lesions as detected on MRI and may reduce the progression of disability [17]. However, despite extensive research it is still not entirely clear how IFNβ exerts its beneficial effects in MS. Treatment with IFNβ in MS has been linked to the inhibition of cell migration [18], down-regulation of cell activation [19], [20], improvement of blood brain barrier (BBB) function [21] and regulation of pro and anti-inflammatory cytokine balance, including IL-1β [22], [23].

Here we show that αCD3-activated human CD4+CD45RO+ memory T-cells primed with IFNβ inhibit pro-IL-1β production and suppress P2X7R-mediated NLRP3 inflammasome activation in a FasL dependent manner. Activated human CD4+CD45RO+ memory T-cells alone inhibited P2X7R-mediated NLRP3 inflammasome activation, but concomitantly increased pro-IL-1β production with a net effect of unchanged active IL-1β release. Priming with IFNβ however unmasked the inhibitory effect on NLRP3 inflammasome activation by additionally reducing pro-IL-1β production. Activated IFNβ–primed CD4+CD45RO+ memory T-cells from multiple sclerosis (MS) patients were not as effective in suppressing NLRP3 inflammasome activation as compared to healthy controls. However memory T-cells from MS patients treated with IFNβwere as suppressive as memory T-cells from healthy controls. Thus, our data demonstrate that human NLRP3 inflammasome is regulated by activated CD4+CD45RO+ memory T cells, and provides new information on the mechanisms mediating the therapeutic effects of IFNβ in MS.

## Materials and Methods

### Subjects

Peripheral blood was obtained after receipt of written informed consent from healthy subjects and MS patients. The study was approved by the institutional review board at Brigham and Women’s hospital for the study of human blood (FWA 00000484). All patients were seen at the Partners Multiple Sclerosis Center at Brigham and Women’s Hospital. MS patients consisted of a group of untreated relapsing-remitting MS patients that had not received steroids in the last 3 months prior to blood drawing, nor IFNβ in the 10 months prior to blood drawing, nor immunosuppressive therapy in the 3 years prior to blood drawing, as well as a group of IFNβ–treated patients. None of the patients were treated with glatiramer acetate prior to blood drawing.

### Reagents

The following human antibodies were used: purified CD3-specific (Biolegend), neutralizing IL10-specific, neutralizing IFNγ–specific (BD Bioscience), neutralizing OX40L-specific, neutralizing CD40L-specific, neutralizing polyclonal FasL-specific, mouse IgG1b-Isotype (RnD Systems), APC-CD4-specific, PE-CD14-specific (Biolegend), FITC-FasL-specific (Abcam), purified caspase-1-specific (RnD Systems). Recombinant active interferon-β was purchased from PBL Interferon Source. Ultrapure LPS was purchased from Invivogen. recFASL and ATP were purchased from SIGMA-Aldrich. P2X7R antagonists KN-62 and AZ 11645373 as well as BzATP were purchased from Tocris Bioscience.

### Cell Isolation and Stimulation of Cytokine Production

Peripheral blood mononuclear cells (PBMC) were obtained by density gradient centrifugation of human peripheral blood over Ficoll-Paque Plus (Amersham Pharmacia Biotech, Uppsala, Sweden).

Monocytes were isolated by CD14+ microbeads (Miltenyi Biotec) according to the manufacturer’s instructions. CD4+CD45RO+ memory T-cells were further negatively enriched from the elution by depletion of non-T-memory cells and CD4+CD45RA+ T-cells (Miltenyi Biotec).

Monocytes (50,000/well in all experiments except where indicated) were plated into a 96-well cell culture plate and allowed to adhere for 2–3 h after which medium (RPMI1640 containing 10%FCS, 10 mM Hepes, 2 mM L-glutamin, 100 U/ml Pencillin and 100 ug/ml Streptomycin) was replaced by medium containing different stimuli and/or CD4+CD45RO+ memory T-cells (100,000/well in all experiments except where indicated). The cells were co-incubated overnight and 100 ng/ml ultrapure LPS was added the following morning for 4 h and subsequently 500 uM ATP for 45 min.

### Cytokine Assays

Levels of IL-1β, pro-IL-1β, sFasL, TNFα, sCaspase–1 and IL-10 in culture supernatants or serum were determined by ELISA using commercial kits from Biolegend (IL-1β and TNFα), RnD Systems (sFasL, pro-IL-1β and Caspase-1) and BD Bioscience (IL-10) following the manufacturer’s instructions.

### Intracellular Cytokine Measurement

Flow cytometric analysis was conducted as described previously [24]. Samples were acquired on FACSCalibur or LSR II flow cytometers (BD Beckinson). Data analysis was performed using the FloJo software (Orlando, FL, USA).

### Immunoblotting

For immunoblotting cells were lysed with RIPA buffer (20 mM Tris–HCl pH 7.4,150 mM NaCl, 1 mM MgCl2, 1 mM CaCl2, 1% Triton X-100) supplemented with protease inhibitor cocktail (Sigma). Lysates were centrifuged to remove particulate matters, resolved on 4–12% Bis-Tris Nupage gels (Invitrogen) and transferred onto PVDF membranes (Millipore). Membranes were blocked for 1 h at room temperature in Tris-buffered saline containing 0.05% Tween 20 (TBST) and 5% dry-milk powder and were subsequently incubated with primary (in TBST with 5% BSA) and appropriate secondary antibodies (in TBST with 5% dry-milk powder). Proteins were visualized using SuperSignal West Femto Maximum Sensitivity Substrate, as instructed by the manufacturer (Pierce).

### Size Exclusion Chromatography

Fractions were achieved by running supernatant on a Superdex 75 prep grade 20×500 mm column (GE Healthcare, Little Chalfont, UK). 500 ul of 10× concentrated supernatant was injected onto the columns and eluted with 50 mm ammonium acetate pH 8.5. An Amersham AKTA fast protein liquid chromatograph (FPLC) (Amersham Biosciences, Piscataway, NJ, USA) was used to collect 1 ml fractions, which were lyophilized and resuspended in complete medium.

### Microarray Analysis

RNA integrity was confirmed using an Agilent 2100 Bioanalyzer (Agilent, Palo Alto, CA) and total RNA samples where then further processed and hybridized to Human Gene ST1.0 Arrays (Affymetrix) according to manufacturer’s instructions. Microarray data from biological replicates were combined and normalized using Nexus. Student’s t-test was used to test for significant differences between gene expression levels of different samples. A correction for the false discovery rate (FDR) was not used in this pair-wise comparison. Only genes that were significantly modulated (p value <0.01) and passed a fold change of FC >2.0 were implemented in differential gene expression signatures and considered for further analysis. All data are MIAME compliant and raw data were submitted to the MIAME compliant database GEO.

**Figure 1:**
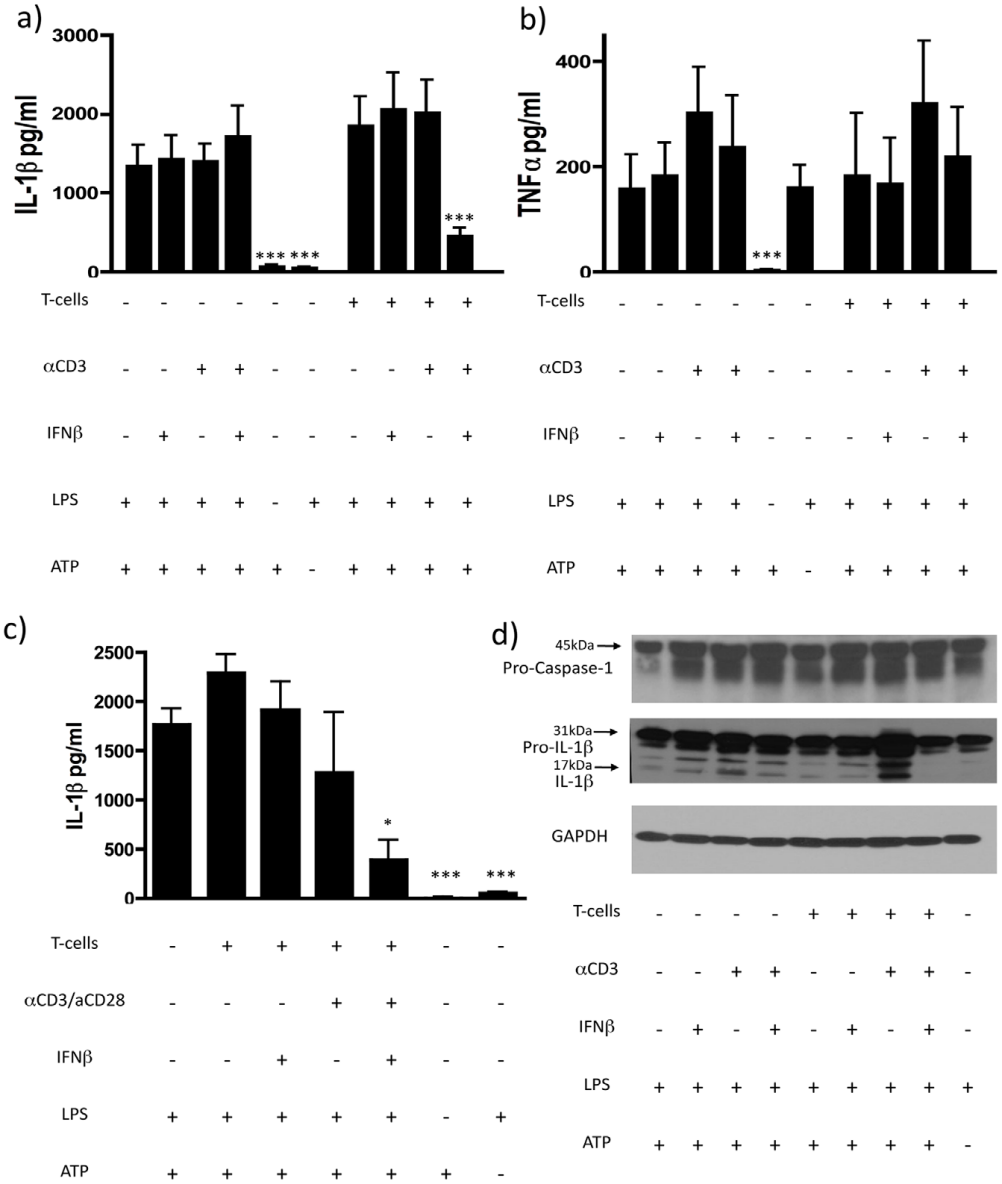
IFNβ inhibits IL-1β release in the presence of activated human CD4+CD45RO+ memory T-cells. Monocytes were incubated overnight in the absence or presence of unstimulated or soluble αCD3 (0.5 ug/ml) activated CD4+CD45RO+CD45RA- memory T-cells with or without 1000 IU/ml IFNβ. The next day 100 ng/ml LPS was added to the co-culture for 4 h, followed by addition of 500 uM ATP for 45 min; a) IL-1β (n = 8) and b) TNFα levels (n = 5) were measured in the supernatant by ELISA. There was a significant reduction of IL-1β release in the presence of activated T-cells and IFNβ; TNFα secretion was not affected; c) memory T-cells were incubated with IFNβ either in the presence or absence of 2 ug/ml αCD3 and αCD28 for 18 h, washed twice with PBS and co-incubated with monocytes for 14 h. The release of IL-1β by monocytes after stimulation with LPS and ATP was suppressed by co-incubation with activated IFNβ-primed memory T-cells (n = 3) (Data are shown as means ± SD of duplicate cultures; *p<0.05; ***p<0.001 employing repeated measures ANOVA with post-hoc Bonferroni adjustment for multiple comparisons to avoid random correlations); d) immunoblot of pro-IL-1β cleavage and pro-caspase-1 levels in the cell lysate of monocytes.

### Quantitative RT-PCR

Total RNA was isolated using the RNeasy micro Kit (Qiagen) and reverse transcribed with the High Capacity cDNA Transcription Kit (Applied Biosystems). Quantitative RTPCR was performed on a 7500 Fast Real-Time PCR System (Applied Biosystems). Customized primers and probes were obtained from Applied Biosystems.

### LDH Cytotoxicity Assay

LDH released from the cells was measured according to manufacturer’s instructions (CytoTox 96® Non-Radioactive Cytotoxicity Assay, Promega). Briefly, cell supernatant was measured for LDH reactivity by enzymatic reaction and measured with a Tecan Infinite F200 microplate reader.

### Intracellular Ca^2+^ Concentration Assay

Intracellular Ca^2+^ concentrations were measured using the compound Fluo4 (Invitrogen) according to manufacturer’s instructions. Briefly, cells were loaded with the calcium detecting dye for 1 h before measurement of intracellular Ca^2+^ concentrations upon stimulation with ATP with a FACSCalibur flow cytometer (BD Beckinson).

### Statistics

Data were analyzed by one-way Anova followed by Bonferroni post-test using Prism 4.0 or paired t-test where applicable.

Differences were considered statistically significant at p<0.05.

## Results

### IFNβ Treated Activated CD4+CD45RO+ Memory T-cells Suppress NLRP3 Inflammasome Activation and Active IL-1β Release by Monocytes

The NLR family, pyrin domain containing 3 (NLRP3) inflammasome activates caspase-1, which then cleaves pro-IL-1β to IL-1β; thus this signaling pathway is an important player in the pathogenesis of both MS and EAE [11], [12], [25]. IFNβ, a first-line therapy for relapsing-remitting MS (RRMS), has been shown to reduce IL-1β secretion by PBMCs. Based on the importance of NLRP3 for the production of mature IL-1β [5], we studied the effect of IFNβ on the activation of the NLRP3 inflammasome in monocytes. We used an *in vitro* system to study the regulation of active IL-1β by IFNβ. Human monocytes were pre-incubated overnight with IFNβ or medium, LPS was added to induce the production of pro-IL-1β and the NLRP3 inflammasome was activated by addition of ATP. Preincubation with IFNβ prior to the addition of LPS and ATP did not affect the release of IL-1β (Fig 1a), suggesting that IFNβ does not act directly on monocytes to regulate the production of active IL-1β.

**Figure 2:**
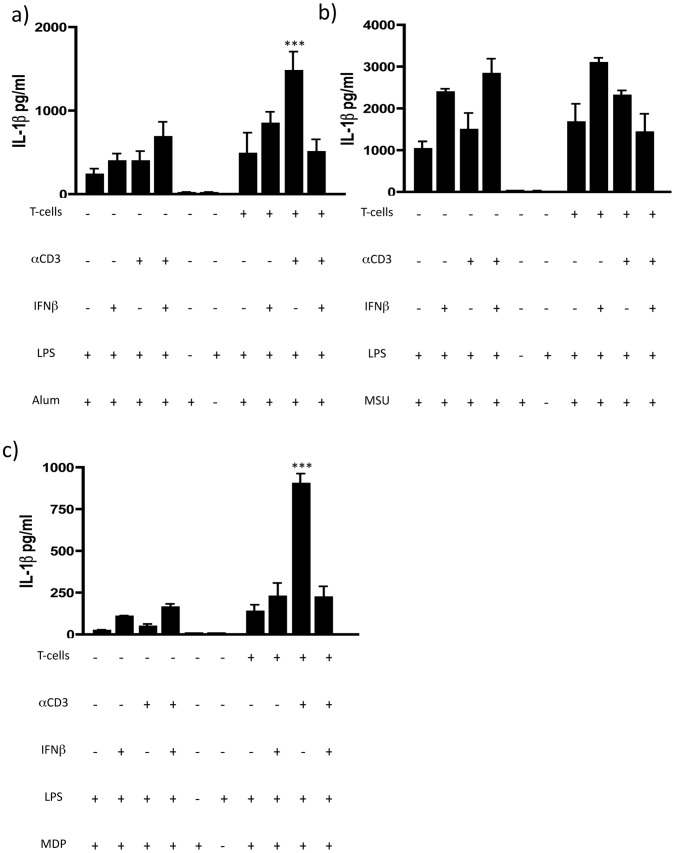
Inhibition of NLRP3 inflammasome activation by IFNβ-primed human CD4+CD45RO+ memory T-cells is ATP-specific. IL-1β release after incubation with a) Alum (400 ug/ml) (n = 3), b) MSU crystals (300 ug/ml) (n = 3) and c) MDP (10 ug/ml) (n = 3) was not inhibited by co-incubation of monocytes with IFNβ-primed CD4+CD45RO+ memory T-cells (Data are shown as means ± SD of duplicate cultures; ***p<0.01 employing repeated measures ANOVA with post-hoc Bonferroni adjustment for multiple comparisons to avoid random correlations).

Murine memory T-cells inhibit the activation of the NLRP1 and NLRP3 inflammasome in a contact dependent manner through the engagement of tumor necrosis factor receptors (TNFR) [26]. Thus, we studied whether IFNβ triggers T-cell-dependent mechanisms that inhibit inflammasome activation in monocytes. We first studied the effect of activated human CD4+CD45RO+CD45RA- memory T-cells on the release of IL-1β by human macrophages. In contrast to the findings of Guarda et al in murine cells [26], we found that the co-incubation of monocytes with human CD4+ memory T cells had no effect on the release of IL-1β by human monocytes (Fig 1a). However, the addition of exogenous IFNβ to the CD4+ memory T-cell monocyte (TM) co-culture led to a marked decrease in the release of mature IL-1β (Fig 1a), similar effects were found when the cleavage of pro-IL-1β was analyzed by WB (Fig 1d). In contrast, no inhibitory effect of IFNβ-primed memory T-cells was detected on the secretion of TNFα (Fig 1b). Analogous to memory T-cells, naïve CD4+CD45RA+CD45RO-T-cells and regulatory CD4+CD25+ T-cells were analysed for their inhibitory effect on secretion of IL-1β by monocytes. Naïve T-cells had a comparable, though somewhat reduced inhibitory effect on monocytes, whereas regulatory T-cells did not have an effect on the secretion of IL-1β (Fig. S1).

To rule out the possibility of a direct effect of IFNβ on monocytes, we preincubated CD4+ memory T-cells overnight with plate-bound αCD3 and αCD28 antibodies in the presence of IFNβ, removed IFNβ by extensive washing and co-incubated the pre-treated T-cells with monocytes for 14 hours. IL-1β release by monocytes after LPS and ATP stimulation was again decreased after co-incubation with IFNβ-primed activated CD4+ memory T-cells (Fig 1c).

**Figure 3:**
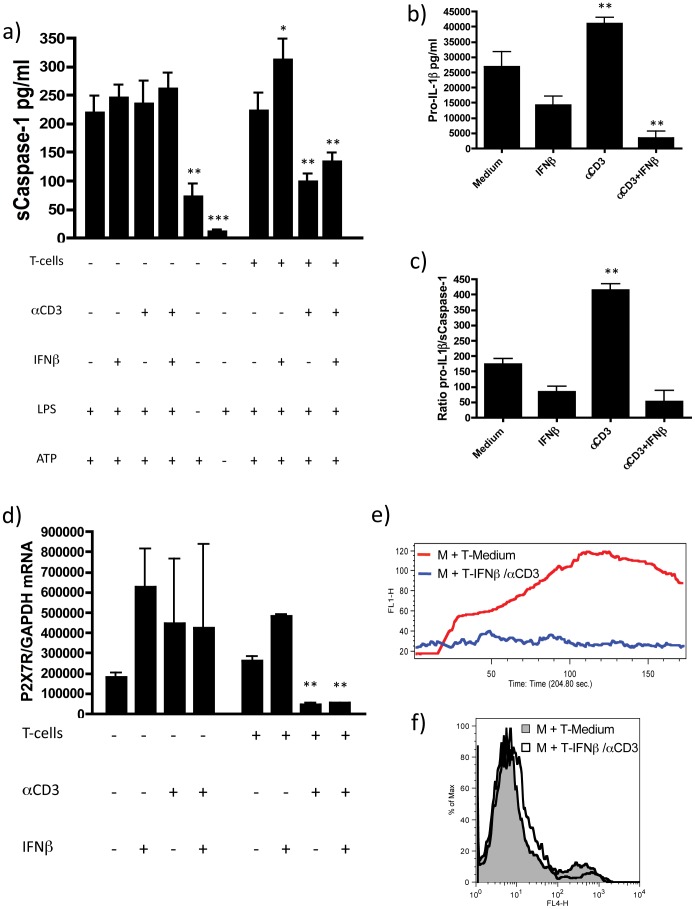
Activated CD4+ CD45RO+ memory T-cells decrease secretion of soluble caspase-1 and P2X7R mRNA expression in monocytes leading to a reduced response upon ATP binding. a) sCaspase-1 levels in the supernatant of monocytes was measured by ELISA (n = 8). A significant decrease of soluble Caspase-1 in the supernatant of cells co-cultured with activated memory T-cells was observed both in the presence and absence of IFNβ; b) intracellular levels of pro-IL-1β measured with an ELISA specific for the immature precursor of IL-1β showed a significant increase in pro-IL-1β levels in the presence of human activated CD4+CD45RO+ memory T-cells (n = 3), cell lysates from adherent monocytes were obtained after removal of non-adherent T-cells after addition of LPS and prior to incubation with ATP c) the ratio of intracellular pro-IL-1β to released soluble Caspase-1 is significantly increased in the presence of human activated CD4+CD45RO+ memory T-cells (n = 3). d) *P2X7R* mRNA expression in monocytes cultured overnight with activated CD4+CD45RO+ memory T-cells is decreased with and without addition of exogenous IFNβ (n = 3), mRNA was isolated from adherent monocytes after removal of non-adherent T-cells after addition of LPS and prior to incubation with ATP (Data are shown as means ± SD of duplicate cultures; *p<0.05; **p<0.01 employing repeated measures ANOVA with post-hoc Bonferroni adjustment for multiple comparisons to avoid random correlations); e) Ca^2+^-influx measurement in monocytes cultured with activated human CD4+CD45RO+ memory T-cells in the presence of IFNβ demonstrates the absence of slow sustained Ca^2+^-influx mediated by ATP binding to the purinergic P2X7-receptor (representative of 3 independent experiments); f) Annexin V staining of monocytes coincubated with T-cells and stimulated with LPS was measured by flow cytometry (representative flow cytometry histogram of n = 5).

**Figure 4:**
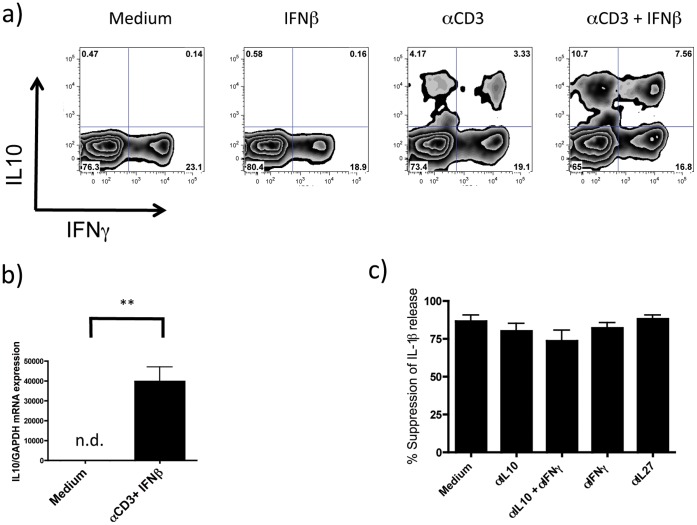
IL10 does not mediate the observed suppression of active IL-1β release. a) memory CD4+CD45RO+ T-cells cultured overnight in the presence of monocytes with or without αCD3 and/or IFNβ were restimulated with PMA/Ionomycin in the presence of Brefeldin A for 4 h and stained for IL-10 and IFNγ (representative of 3 independent experiments); b) upregulation of *IL10* mRNA expression in resorted memory T-cells was confirmed by qPCR (n = 3; n.d. = not detected); c) inhibition of IL-10 (10 ug/ml) and/or IFNγ (10 ug/ml) by specific blocking antibodies did not affect the suppressive effect on IL-1β release by activated T-cells in the presence of IFNβ (n = 4) (% suppression is calculated by subtracting from 1 the ratio of IL-1β release from activated T-cells in the presence of IFNβ and non-activated T-cells in the absence of IFNβ and multiplying by 100; **p<0.01 employing repeated measures ANOVA with post-hoc Bonferroni adjustment for multiple comparisons to avoid random correlations).

NLRP3 inflammasome can be activated by various other signals such as Alum or MSU crystals that involve unspecific membrane disruption rather than P2X7R activation. To investigate whether NLRP3 inflammasome inactivation was confined to ATP mediated ligation of P2X7R we tested other P2X7R-independent NLRP3 inflammasome activators. Surprisingly, neither stimulation with Alum (Fig 2a) nor with monosodium urate (MSU) crystals (Fig 2b) was inhibited by IFNβ–primed memory T-cells. Also NLRP1 inflammasome activation by MDP was not inhibited by incubation with IFNβ-primed memory T-cells (Fig 2c), suggesting that the inhibitory effect of IFNβ-primed activated CD4+CD45RO+ memory T-cells on pro-IL-1β cleavage is specific to ATP-mediated P2X7R-activation. Thus, IFNβ-primed memory T-cells inhibit ATP-triggered NLRP3 inflammasome activation and the release of active IL-1β by monocytes.

### Soluble Caspase-1 Release is Decreased in Monocytes Cultured with Activated CD4+CD45RO+ Memory T-cells

Upon activation, the NLRP3 inflammasome activates caspase-1, which then cleaves pro-IL-1β to mature IL-1β. Therefore, inflammasome activation is reflected by increased cellular levels of active caspase-1, which is rapidly secreted from the cells [5]. Our data suggested the post-transcriptional regulation of IL-1β release by IFNβ-primed memory T-cells, thus we investigated the effect that co-culture of IFNβ-treated CD4+ memory T-cells with monocytes might have on inflammasome activity and the production of soluble caspase-1 (sCaspase-1). We found that sCaspase-1 levels were decreased in the presence of activated T-cells, with or without the addition of IFNβ (Fig 3a). This observation seemed contradictory, as this decrease in the levels of sCaspase-1 in the co-culture with αCD3 activated T-cells was not mirrored by a decrease in IL-1β secretion. However, when we measured intracellular levels of pro-IL-1β in the different culture conditions we found a marked upregulation in the levels of pro-IL-1β in monocytes co-cultured with activated T-cells (Fig 3b). Thus, the ratio of pro-IL-1β to sCaspase-1 was significantly increased in the co-culture of activated CD4+ memory T-cells and monocytes suggesting that the release of mature IL-1β is controlled not only by the availability of active caspase-1, but also by the concentration of pro-IL-1β in the cells (Fig 3c).

The purinergic receptor P2X7R triggers the assembly of the NLRP3 inflammasome in response to ATP [27], [28]. We found that the decrease in sCaspase-1 levels in the supernatants was associated with a decrease in *P2X7R* mRNA expression (Fig 3d). Upon ATP binding, P2X7R mediates a slow sustained influx of extracellular calcium that follows an initial fast efflux of intracellular calcium stores mediated by the purinergic receptor P2Y [29]. Thus, P2X7R function can be investigated employing a functional calcium flux assay [30]. Monocytes co-incubated with activated CD4+CD45+ memory T-cells in the presence of IFNβ showed a significant reduction in calcium influx (Fig 3e) suggesting that IFNβ-primed activated CD4+CD45RO+ memory T-cells control the response to ATP. Addition of P2X7R specific inhibitors led to a comparable inhibition of Ca^2+^-influx (Fig. S2) further validating the applicability of the assay to measure inihibition of P2X7R-induced Ca^2+^-influx. Stimulation of monocytes with the P2X7R specific agonist BzATP led to a comparable Ca^2+^-influx that was also inhibited by addition of memory T-cells in the presence of αCD3 and IFNβ (Fig. S3). In summary, activated CD4+CD45RO+ memory T-cells suppress ATP-triggered NLRP3 inflammasome activation in monocytes by the inhibition of P2X7R-mediated signaling.

**Figure 5:**
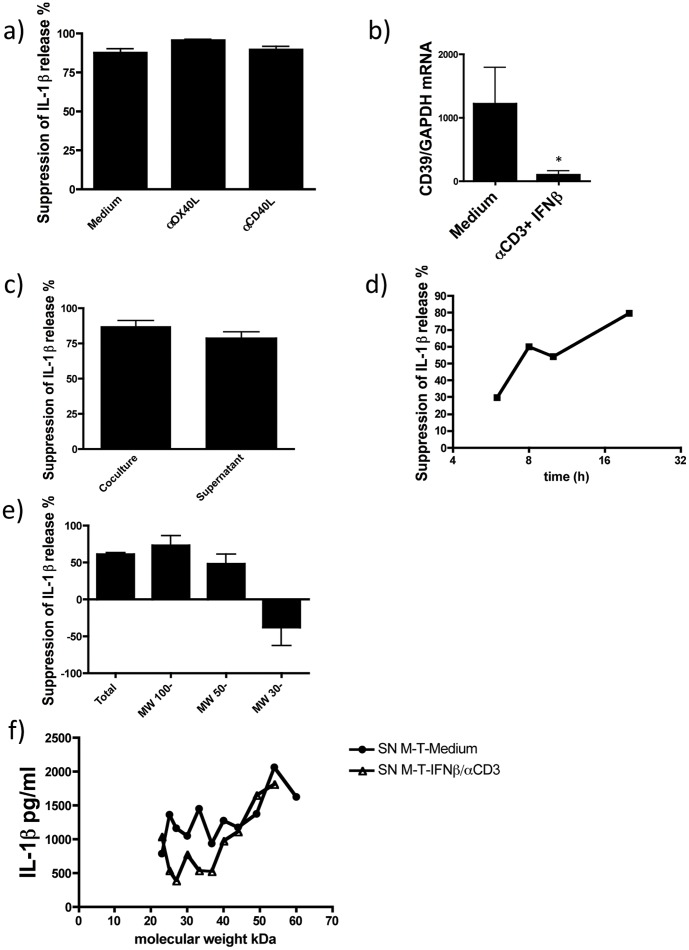
Suppression of IL-1β release is cell contact independent and mediated by a soluble factor of 23–38 kDa MW. a) Blocking OX40L and CD40L pathways by the use of inhibitory antibodies did not affect the suppressive effect of activated T-cells on monocytes in the presence of IFNβ (n = 3; % suppression calculated as described in Fig 4); b) *CD39* mRNA expression by memory T-cells resorted after co-culture with monocytes was measured by qPCR (n = 3) (Data are shown as means ± SD of duplicate cultures; *p<0.01 employing repeated measures ANOVA with post-hoc Bonferroni adjustment for multiple comparisons to avoid random correlations); c) transfer of supernatant conditioned by a co-culture of monocytes and activated memory CD4+ T-cells in the presence of IFNβ onto fresh monocytes reproduced the suppressive effect on IL-1β release (n = 4); d) Kinetics of the inhibitory factor release and determination of molecular weight; supernatants of monocytes co-cultured with activated human memory T-cells in the presence of IFNβ were removed at the indicated time-points and fresh monocytes were cultured in the conditioned supernatants for 16 h before stimulation with LPS and activation with ATP (representative of 2 independent experiments); e) supernatants were filtered through molecular cut-off filters or f) SEC fractionated and molecular weight fractions in a 1∶1 ratio with fresh medium were used for the culture of fresh monocytes. IL-1β secretion was measured and inhibition of release was calculated according to Fig 4 (representative of 2 independent experiments).

### The Inhibition of Active IL-1β Release is Independent of IL-10

Treatment with IFNβ leads to an increase in the levels of the anti-inflammatory cytokine IL-10 in the circulation [31]. Thus, we analyzed the role of IL-10 in the inhibition of IL-1β release by IFNβ. Intracellular cytokine staining showed an increase in IL-10 and IFNγ positive T-cells in response to *in vitro* IFNβ treatment (Fig 4a). Similarly, we also found an upregulation in *IL10* mRNA expression in resorted activated T-cells that were cultured in the presence of IFNβ and monocytes (Fig 4b).

We thus investigated the role of IL-10 in the control of IL-1β release by IFNβ-treated memory T cells. The addition of exogenous recombinant IL-10 suppressed IL-1β production, and this suppression was abrogated by addition of IL-10 blocking antibody (Fig. S4). Strikingly, although IL-10 suppressed IL-1β release, validated blocking antibodies to IL-10 had no effect on the suppression of IL-1β release by IFNβ (Fig 4c). Furthermore analysis of mRNA expression revealed inhibition of *IL1B* transcription by monocytes co-incubated with T-cells in the presence of αCD3 and IFNβ which was inhibited by co-incubation with an IL-10-blocking antibody (Fig.S5). Thus, although IL-10 inhibits *IL1B* expression at the transcriptional level [32], IFNβ also triggers IL-10 independent post-transcriptional regulatory mechanisms that suppress the release of active IL-1β by monocytes.

**Figure 6:**
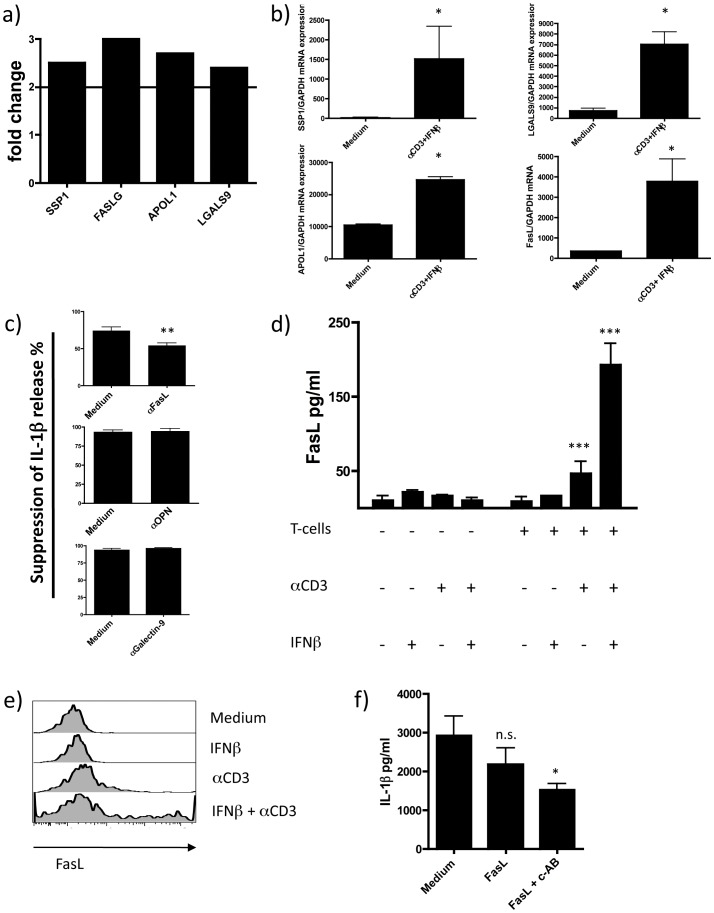
Identification of sFasL mediating the inhibition of IL-1β release. a) Microarray analysis was performed in memory T-cells cultured in the presence of monocytes with and without IFNβ for 16 h and resorted by gating on CD3+CD4+7AAD- cells, upregulated candidates that fit the criteria of presence of a soluble form and molecular weight of 23–38 kDa are displayed; b) qPCR confirmation of upregulation of SSP1, LGALS9, APOL1 and FasL mRNA expression through incubation with IFNβ (n = 3); c) analysis of biological relevance of upregulated molecules by the use of blocking antibodies (10 ug/ml), only blockage of FasL significantly decreased the inhibitory effect on IL-1β release (n = 3) (% suppression calculated as described in Fig 4); d) soluble FasL levels were measured with a FasL specific ELISA in the supernatants of co-cultured activated CD4+CD45RO+ memory T-cells (n = 3); e) surface expression of FasL is measured after overnight co-culture by staining with FasL specific antibody and acquisition of cells by flow cytometry (representative of 3 independent experiments); f) IL-1β release is inhibited by recFasL in the presence of a crosslinking antibody (n = 3) (Data are shown as means ± SD of duplicate cultures; *p<0.05, **p<0.01, ***p<0.001 employing repeated measures ANOVA with post-hoc Bonferroni adjustment for multiple comparisons to avoid random correlations).

**Figure 7:**
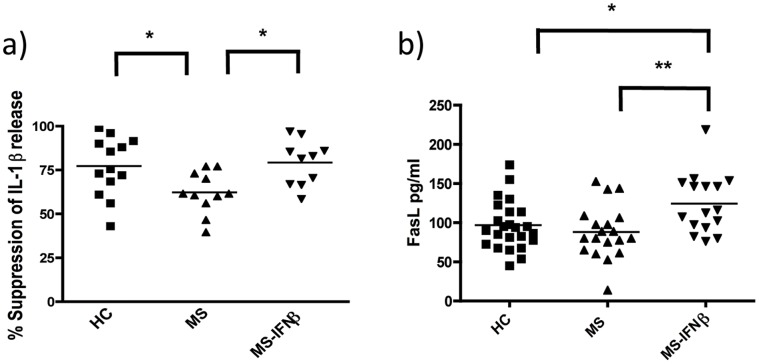
CD4+CD45RO+ memory T-cells of untreated MS patients are defective in the IFNβ-induced suppressive ability on active IL-1β release by monocytes. This suppressive ability is rescued by *in vivo* treatment with IFNβ and is associated with increased serum levels of sFasL in IFNβ-treated MS patients; a) monocytes of HC (healthy controls) were cultured overnight with allogenic activated memory T-cells from HC (n = 13), untreated (n = 11) or IFNβ-treated MS patients (n = 10) and the suppressive effect of exogenous IFNβ on IL-1β release was measured as described in Fig 4; activated memory T-cells of MS patients showed a significantly decreased suppressive effect when compared to healthy controls, which was reversed by *in vivo* treatment with IFNβ; b) FasL levels in the serum of healthy controls (HC) (n = 24), untreated MS patients (MS) (n = 19) and IFNβ-treated MS patients (MS-IFNβ) (n = 16) were measured by ELISA; IFNβ treated MS patients showed an increased level of sFasL when compared to untreated MS patients and healthy controls; (*p<0.05; **p<0.01 employing repeated measures ANOVA with post-hoc Bonferroni adjustment for multiple comparisons to avoid random correlations).

### The Suppression of Inflammasome Activation is Mediated by a Soluble Factor

We next analyzed whether the inhibitory effect of CD4+ memory T-cells is contact-dependent and mediated by TNFR family members as reported in murine cells [26]. Blocking antibodies specific for the TNFR family members CD40L and OX40L did not affect the suppression of IL-1β release by IFNβ-treated memory T-cells (Fig 5a). Furthermore T-cells can upregulate the surface molecule CD39 an ecto-enzyme that hydrolyzes ATP to ADP. We tested whether upregulation of CD39 by αCD3 activation and IFNβ stimulation could contribute to the inhibitory effect on IL-1β secretion by monocytes. mRNA of FACS-resorted CD4+CD45RO+ memory T-cells was isolated and relative mRNA expression of *CD39* was determined. However *CD39* mRNA expression was decreased in T-cells after stimulation with αCD3 and IFNβ in the T-cell monocyte co-culture (Fig 5b), thus CD39 does not seem to be involved in the suppression of IL-1β release.

In further experiments we incubated monocytes with supernatant conditioned by a co-culture of monocytes and memory T-cells and observed a comparable inhibitory effect (Fig 5c), suggesting that the suppression of IL-1β release in monocytes is mediated by a soluble factor.

Next we analyzed the kinetics of the production of the factor responsible for the inhibition of IL-1β release. Fresh monocytes were incubated overnight with supernatants taken at different time-points from co-cultures of memory T-cells and monocytes. We found a direct correlation between increasing time of co-culture and inhibition of active IL-1β secretion (Fig 5d). To estimate the molecular weight of the soluble factor that mediates the suppression of inflammasome activation by CD4+CD45RO+ memory T-cells we used molecular weight cut-off filters. Supernatants were fractioned with molecular weight cut-off filters of 100, 50 and 30 kDa and the flow-throughs were collected and cultured with fresh monocytes. The factor responsible for the inhibition of IL-1β release was still present when flow-throughs of 100 kDa, and 50 kDa cut-off filters were tested on monocytes, but not when the flow-through of 30 kDa cut-off filters was used (Fig 5e). Thus, we estimated the molecular weight of the IL-1β release inhibitory factor to be in the range of 30–50 kDa. Size exclusion chromatography further confirmed and narrowed down the molecular weight range to 23–38 kDa (Fig 5f – of note the observed difference of inhibition in high molecular weight fractions was thought secondary to high molecular weight of nutrients such as BSA). In conclusion, memory T-cells co-cultured with monocytes in the presence of IFNβand αCD3 release a soluble factor of 23–38 kDa that inhibits inflammasome activation and the secretion of active IL-1β.

### FasL Contributes to the Inhibition of IL-1β Release by Monocytes

To identify the soluble factor responsible for the decreased release of IL-1β we performed a microarray analysis. Live CD3+ CD4+ T-cells from the monocyte T-cell co-culture were re-isolated by FACS sorting and gene expression was analyzed with Gene ST1.0 microarrays (Affymetrix). We then used three criteria to select candidate molecules mediating inflammasome inhibition in monocytes: 1) upregulation by co-culture of memory T-cells with monocytes and IFNβ, 2) molecular weight between 23–38 kDa and 3) existence of a soluble form. We identified FasL, LGALS9, APOL1 and SPP1 as matching candidates (Fig 6a). We confirmed the upregulation of these candidates in T-cell-monocyte co-cultures by qPCR (Fig 6b). To investigate the biological relevance of these candidates we used blocking antibodies specific for the identified candidates or purified APOL1. We found that only FasL was involved in the inhibitory effect of IFNβ-treated memory T-cells on IL-1β release, as addition of FasL blocking antibodies diminished the suppression of IL-1β release (Fig 6c). However, as the inhibition was not completely reversed, another yet unidentified soluble factor seems to be involved. Expression of FasL on the cell surface was demonstrated by flow cytometry and an increase in soluble FasL (sFasL) in the supernatant of the co-culture was shown by ELISA (Fig 6d and e). Addition of recFasL led to an inhibition of IL-1β release itself, however not statistically significant, further crosslinking of recFasL by addition of tag-specific antibody led to a significant decrease of IL-1β release (Fig 6f). Of note, a potential increase in apoptosis was ruled out by measurement of LDH release in the supernatant (Fig. S6). Thus, IFNβ triggers the secretion of FasL by human CD4+ CD45RO+ memory T-cells, which then contributes to the inhibition of the release of mature IL-1β by monocytes.

### Influence of IFNβ Treatment on Inflammasome Activation in MS Patients

IL-1β in combination with IL-21, TGFβ and IL-23, promotes the differentiation and expansion of Th17 cells [7], [8], [9], [10], [33], which are thought to play an important role in the pathogenesis of MS [34]. Increased frequencies of Th17 cells have been found in MS patients [35]. Thus, we studied whether the suppressive activity of CD4+CD45RO+ memory T cells on IL-1β secretion was impaired in MS patients. CD4+CD45RO+ memory T-cells of MS patients were isolated and co-cultured with monocytes from healthy controls. We found that the suppressive activity of CD4+CD45RO+ memory T-cells on IL-1β secretion by monocytes was significantly reduced in untreated MS patients compared to healthy controls (p<0.05) (Fig 7a). Treatment with IFNβ reversed this reduced suppressive effect as CD4+ memory T-cells of IFNβ-treated MS patients showed a comparable suppressive activity to healthy controls (Fig 6a). Having found that IFNβ–induced sFasL contributes to the down-regulation of IL-1β release *in vitro*, we investigated whether FasL induction is associated with reduction of IL-1β secretion in response to the treatment with IFNβ. IFNβ–treated MS patients showed a significant increase in sFasL serum levels as compared to healthy controls and untreated MS patients (Fig 7b). Taken together these data suggest that treatment with IFNβ leads to increased suppressive ability of CD4+CD45RO+ memory T-cells on IL-1β secretion by monocytes partly through an increase in sFasL serum levels.

## Discussion

The secretion of IL-1β is tightly controlled by inflammasomes, multi-protein complexes composed of NLR and adaptor proteins that aggregate upon exposure to specific activators. Owing to their central role in the secretion of active IL-1β, inflammasomes play an important role in the control of the immune response to tumors [36] and infections [37], and also in autoimmune diseases such as MS [38]. Thus, it is important to characterize the regulatory mechanisms that control inflammasome activation. Although it has recently been shown that CD4+ memory T-cells control the activation of murine NLRP3 and NLRP1 inflammasomes [26], the mechanisms controlling human inflammasome activation are largely unknown. Here we show that activated human CD4+CD45RO+ memory T-cells primed with IFNβ suppress pro-IL-1β production and the P2X7R-mediated activation of NLRP3 inflammasome in a partly FasL-dependent manner, through a mechanism different from that involved in the control of murine inflammasomes.

Activated CD4+ memory T cells have been shown to suppress the activation of mouse inflammasomes by mechanisms dependent on ligands of the TNFR family, such as CD40L and RANKL [26]. Our data suggest that human NLRP3 inflammasome is regulated differently. We found that activated human CD4+CD45RO+ memory T cells did not suppress active IL-1β release unless they had been previously pre-activated with IFNβ. Activated human CD4+CD45RO+ memory T-cells increase the production of pro-IL-1β in monocytes, but at the same time downregulate *P2X7R* mRNA expression and consequently, the influx of extracellular calcium following ATP stimulation. Thus these two limiting steps of active IL-1β secretion are counter regulated and eventually lead to a net unchanged secretion of active IL-1β. Priming of activated human CD4+CD45RO+ memory T-cells with IFNβ renders the T–cells able to also inhibit the production of pro-IL-1β in monocytes. Therefore IFNβ-priming unmasks the ability of activated human CD4+CD45RO+ memory T-cells to inhibit ATP-mediated NLRP3 inflammasome activation through a partly FasL-dependent mechanism that is apoptosis-independent [39], [40]. Thus, activated human CD4+CD45RO+ memory T-cells regulate the NLRP3 inflammasome activation in monoyctes by controlling the response of monocytes to ATP.

The activation of NLRP3 inflammasome by P2X7R-independent triggers such as Alum and MSU crystals was not affected by co-incubation with IFNβ-primed activated CD4+CD45RO+ memory T-cells, suggesting that the inhibition of NLRP3 inflammasome activation is restricted to ATP-mediated P2X7R signaling. Previous reports on inhibition of inflammasome activation in murine cells have demonstrated stimulus nonspecific inactivation [26], suggesting inhibition upstream of inflammasome assembly but downstream of stimulus-specific signaling. Thus, this is the first report of a stimulus specific inhibition of inflammasome activation, demonstrating that inflammasome activation can be inhibited in a stimulus-specific manner, without affecting potentially important alternative triggering mechanisms needed to fight off pathogens. ATP is quickly released from cells upon encountering stress such as tissue trauma or infection [41] and P2X7R-mediated neuroinflammation has been previously attributed an important role in the development of EAE [42]. Currently specific CNS-penetrant P2X7R-antagonists are being developed to be used as anti-inflammatory medication in neuroinflammatory diseases such as MS [43] emphasizing the importance of the pro-inflammatory function of ATP-triggered P2X7R activation.

It has recently been reported that type I interferon acts directly on mouse macrophages and human monocytes to inhibit inflammasome activation [32]. Our data do not support a direct effect of IFNβ on human monocytes during the suppression of inflammasome activation. We believe that the discrepancies between our study and that of Guarda et al reflect differences in the regulatory mechanisms that control murine and human inflammasome activation.

IFNβ is a first line therapy for RRMS [14], [15], [16]. We found that IFNβ-primed CD4+CD45RO+ memory T-cells from untreated MS patients have an impaired ability to suppress ATP-mediated NLRP3 inflammasome activation and IL-1β release in monocytes. However, this impaired suppressive activity was not found when we analyzed samples from IFNβ-treated patients. Thus, supplementation of IFNβ *in vivo* might prime CD4+ CD45RO+ memory T-cells to become more responsive to stimulation with IFNβ *in vitro*. Alternatively, a specific subpopulation of CD4+CD45RO+ memory T-cells responsible for the suppression of inflammasome activation in monocytes might be decreased or dysfunctional in untreated MS patients, but recovered following treatment with IFNβ. The innate immune system plays an important role in multiple sclerosis and infections have been shown to trigger MS exacerbations, thus the regulation of TLR and ATP-induced NLRP3 inflammasome activation by IFNβ might contribute to the beneficial effects of IFNβ treatment in MS [44].

The regulation of pro-IL-1β production and NLRP3 inflammasome activation by IFNβ-primed activated CD4+CD45RO+ memory T cells might also play a role in limiting immunopathology during the immune response to viruses. The immune response to viral infections is usually characterized by the production of high levels of IFNβ, which triggers a cascade of anti-viral mechanisms [15]. Our data demonstrate that in addition to its direct anti-viral effects, IFNβ also triggers a negative feedback loop that ultimately arrests inflammasome activation and IL-1β production. In combination with other anti-inflammatory effects of IFNβ such as the inhibition of the differentiation of Th17 cells [45] and the promotion of the differentiation of Tr1 cells [46], the suppression of pro-IL-1β production and inflammasome activation by IFNβ-primed activated CD4+CD45RO+ memory T cells is likely to limit the immune response triggered by infections. Moreover, considering the linkage existing between infections and MS [47], it is possible that deficits in the ability of CD4+CD45RO+ memory T cells to control the immune response triggered by pathogens might play a role in the development of MS.

In conclusion, we describe a new mechanism by which CD4+CD45RO+ memory T cells control human innate immunity. Activated CD4+CD45RO+ memory T-cells primed by IFNβ, which itself is produced by cells of the innate immune system upon activation, down-regulate the production of pro-IL-1β and the response of monocytes to ATP and consequently, P2X7R-mediated NLRP3 inflammasome activation in a partly FasL-dependent manner. This negative feedback loop mediated by human IFNβ and activated CD4+CD45RO+ memory T–cells is likely to limit inflammation and immunopathology during the course of the immune response to viruses, and might contribute to the therapeutic effects of IFNβ in MS.

## Supporting Information

Figure S1 **Effect of naïve and regulatory T-cells on IL-1β release by monocytes.** a) Naïve CD4^+^CD45RO^-^CD45RA^+^ (FACS sorted) T-cells inhibit IL-1β release by monocytes in the presence of αCD3 and IFNβ; b) CD4^+^CD25^+^ regulatory T-cells do not inhibit IL-1β release by monocytes in the presence of αCD3 and IFNβ50.000 cells/well CD4^+^CD25^+^ (FACS-sorted) regulatory T-cells co-incubated with 50.000 cells/well monocytes, a T-cell/monocyte ratio in memory T-cells that is sufficient for inhibition of IL-1β release (not shown)).
(DOCX)

Figure S2 **ATP induced Ca^2+^-influx is inhibited by specific P2X7R-antagonists.** a) Ca^2+^-influx induced by ATP in the absence of inhibitor (red), in the presence of 10 uM KN-62 (blue), in the presence of 100 uM KN-62 (green); b) Ca^2+^-influx induced by ATP in the absence of inhibitor (red), in the presence of 10 uM AZ 11645373 (blue), in the presence of 100 uM AZ 11645373 (green).
(DOCX)

Figure S3 **BzATP induces Ca^2+^-influx comparable to ATP and, which is inhibited by co-incubation with activated T-cells in the presence of IFNβ.** a) ATP induced Ca^2+^-influx (red) is comparable to BzATP induced Ca^2+^-influx (blue); b) BzATP-induced Ca^2+^-influx in the presence of T-cells with (red) or without αCD3 and IFNβ (blue).
(DOCX)

Figure S4 Recombinant IL-10 suppresses IL-1β release by LPS and ATP stimulated monocytes, which is abrogated by a specific IL10-blocking antibody (10 ug/ml) (**p<0.01 employing repeated measures ANOVA with post-hoc Bonferroni adjustment for multiple comparisons to avoid random correlations).
(DOCX)

Figure S5 **mRNA expression of** ***IL1B*** is inhibited by co-incubation of monocytes with T-cells in the presence of αCD3 and IFNβ, this inhibition is reversed by addition of an IL-10 blocking antibody (n = 3; *p<0.05 employing repeated measures ANOVA with post-hoc Bonferroni adjustment for multiple comparisons to avoid random correlations).
(DOCX)

Figure S6 **No differences in extracellular LDH levels were detected in the co-cultures of monocytes with CD4+CD45RO+ memory T-cells (n = 3).**
(DOCX)
