# Supplementary material for: Activated Human CD4+CD45RO+ Memory T-Cells Indirectly Inhibit NLRP3 Inflammasome Activation through Downregulation of P2X7R Signalling
Source: PLoS One. 2012 Jun 29;7(6):e39576. doi: 10.1371/journal.pone.0039576 (PMC3387029; doi:10.1371/journal.pone.0039576)
Supplement: Figure S1 — Effect of naïve and regulatory T-cells on IL-1β release by monocytes. a) Naïve CD4+CD45RO-CD45RA+ (FACS sorted) T-cells inhibit IL-1β release by monocytes in the presence of αCD3 and IFNβ; b) CD4+CD25+ regulatory T-cells do not inhibit IL-1β release by monocytes in the presence of αCD3 and IFNβ50.000 cells/well CD4+CD25+ (FACS-sorted) regulatory T-cells co-incubated with 50.000 cells/well monocytes, a T-cell/monocyte ratio in memory T-cells that is sufficient for inhibition of IL-1β release (not shown)). (DOCX) [file pone.0039576.s001.docx]

a) b)


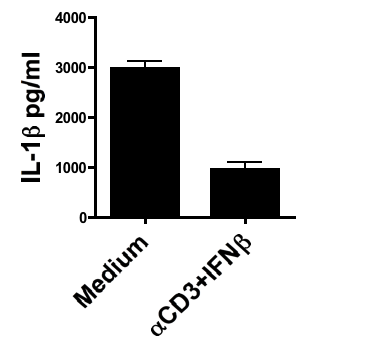

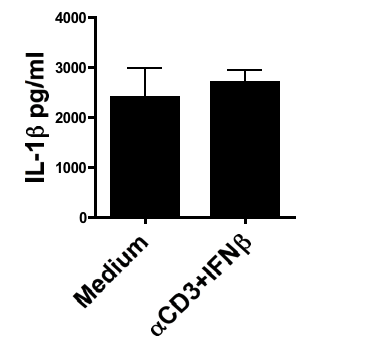


**

**Fig.S1** *Effect of naïve and regulatory T-cells on IL-1β release by monocytes.* a) Naïve CD4^+^CD45RO^-^CD45RA^+^ (FACS sorted) T-cells inhibit IL-1β release by monocytes in the presence of αCD3 and IFNβ; b) CD4^+^CD25^+^ regulatory T-cells do not inhibit IL-1β release by monocytes in the presence of αCD3 and IFNβ 50.000 cells/well CD4^+^CD25^+^ (FACS-sorted) regulatory T-cells co-incubated with 50.000 cells/well monocytes, a T-cell/monocyte ratio in memory T-cells that is sufficient for inhibition of IL-1β release (not shown)).
