# Supplementary material for: Activated Human CD4+CD45RO+ Memory T-Cells Indirectly Inhibit NLRP3 Inflammasome Activation through Downregulation of P2X7R Signalling
Source: PLoS One. 2012 Jun 29;7(6):e39576. doi: 10.1371/journal.pone.0039576 (PMC3387029; doi:10.1371/journal.pone.0039576)
Supplement: Figure S2 — ATP induced Ca2+-influx is inhibited by specific P2X7R-antagonists. a) Ca2+-influx induced by ATP in the absence of inhibitor (red), in the presence of 10 uM KN-62 (blue), in the presence of 100 uM KN-62 (green); b) Ca2+-influx induced by ATP in the absence of inhibitor (red), in the presence of 10 uM AZ 11645373 (blue), in the presence of 100 uM AZ 11645373 (green). (DOCX) [file pone.0039576.s002.docx]

a)


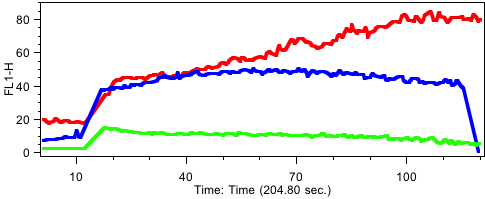


b)


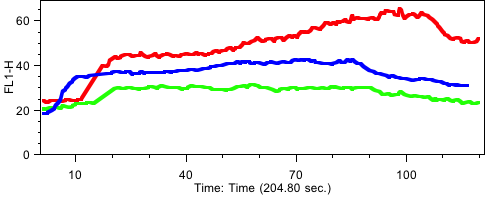


**Fig.S2** *ATP induced Ca^2+^-influx is inhibited by specific P2X7R-antagonists.* a) Ca^2+^-influx induced by ATP in the absence of inhibitor (red), in the presence of 10uM KN-62 (blue), in the presence of 100uM KN-62 (green); b) Ca^2+^-influx induced by ATP in the absence of inhibitor (red), in the presence of 10uM AZ 11645373 (blue), in the presence of 100uM AZ 11645373 (green).
