# Supplementary material for: Activated Human CD4+CD45RO+ Memory T-Cells Indirectly Inhibit NLRP3 Inflammasome Activation through Downregulation of P2X7R Signalling
Source: PLoS One. 2012 Jun 29;7(6):e39576. doi: 10.1371/journal.pone.0039576 (PMC3387029; doi:10.1371/journal.pone.0039576)
Supplement: Figure S3 — BzATP induces Ca2+-influx comparable to ATP and, which is inhibited by co-incubation with activated T-cells in the presence of IFNβ. a) ATP induced Ca2+-influx (red) is comparable to BzATP induced Ca2+-influx (blue); b) BzATP-induced Ca2+-influx in the presence of T-cells with (red) or without αCD3 and IFNβ (blue). (DOCX) [file pone.0039576.s003.docx]

a)


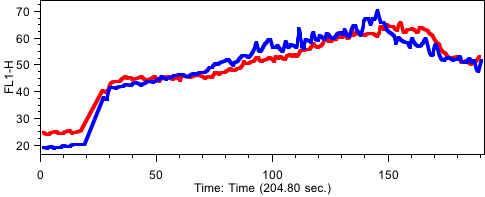


b)


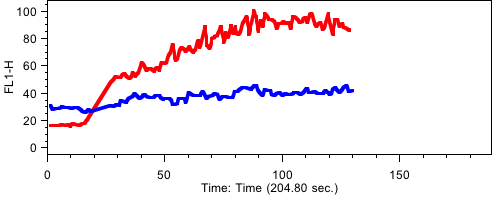


**Fig.S3** BzATP induces Ca^2+^-influx comparable to ATP and, which is inhibited by co-incubation with activated T-cells in the presence of IFNβ; a) ATP induced Ca^2+^-influx (red) is comparable to BzATP induced Ca^2+^-influx (blue); b) BzATP-induced Ca^2+^-influx in the presence of T-cells with (red) or without αCD3 and IFNβ (blue).
