# Supplementary material for: Activated Human CD4+CD45RO+ Memory T-Cells Indirectly Inhibit NLRP3 Inflammasome Activation through Downregulation of P2X7R Signalling
Source: PLoS One. 2012 Jun 29;7(6):e39576. doi: 10.1371/journal.pone.0039576 (PMC3387029; doi:10.1371/journal.pone.0039576)
Supplement: Figure S4 — Recombinant IL-10 suppresses IL-1β release by LPS and ATP stimulated monocytes, which is abrogated by a specific IL10-blocking antibody (10 ug/ml) (**p<0.01 employing repeated measures ANOVA with post-hoc Bonferroni adjustment for multiple comparisons to avoid random correlations). (DOCX) [file pone.0039576.s004.docx]

**Fig.S4** Recombinant IL-10 suppresses IL-1β release by LPS and ATP stimulated monocytes, which is abrogated by a specific IL10-blocking antibody (10ug/ml) (**p<0.01 employing repeated measures ANOVA with post-hoc Bonferroni adjustment for multiple comparisons to avoid random correlations).
