# Supplementary material for: Activated Human CD4+CD45RO+ Memory T-Cells Indirectly Inhibit NLRP3 Inflammasome Activation through Downregulation of P2X7R Signalling
Source: PLoS One. 2012 Jun 29;7(6):e39576. doi: 10.1371/journal.pone.0039576 (PMC3387029; doi:10.1371/journal.pone.0039576)
Supplement: Figure S5 — mRNA expression of IL1B is inhibited by co-incubation of monocytes with T-cells in the presence of αCD3 and IFNβ, this inhibition is reversed by addition of an IL-10 blocking antibody (n = 3; *p<0.05 employing repeated measures ANOVA with post-hoc Bonferroni adjustment for multiple comparisons to avoid random correlations). (DOCX) [file pone.0039576.s005.docx]

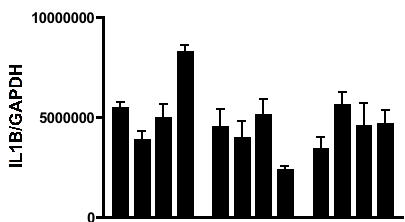


*


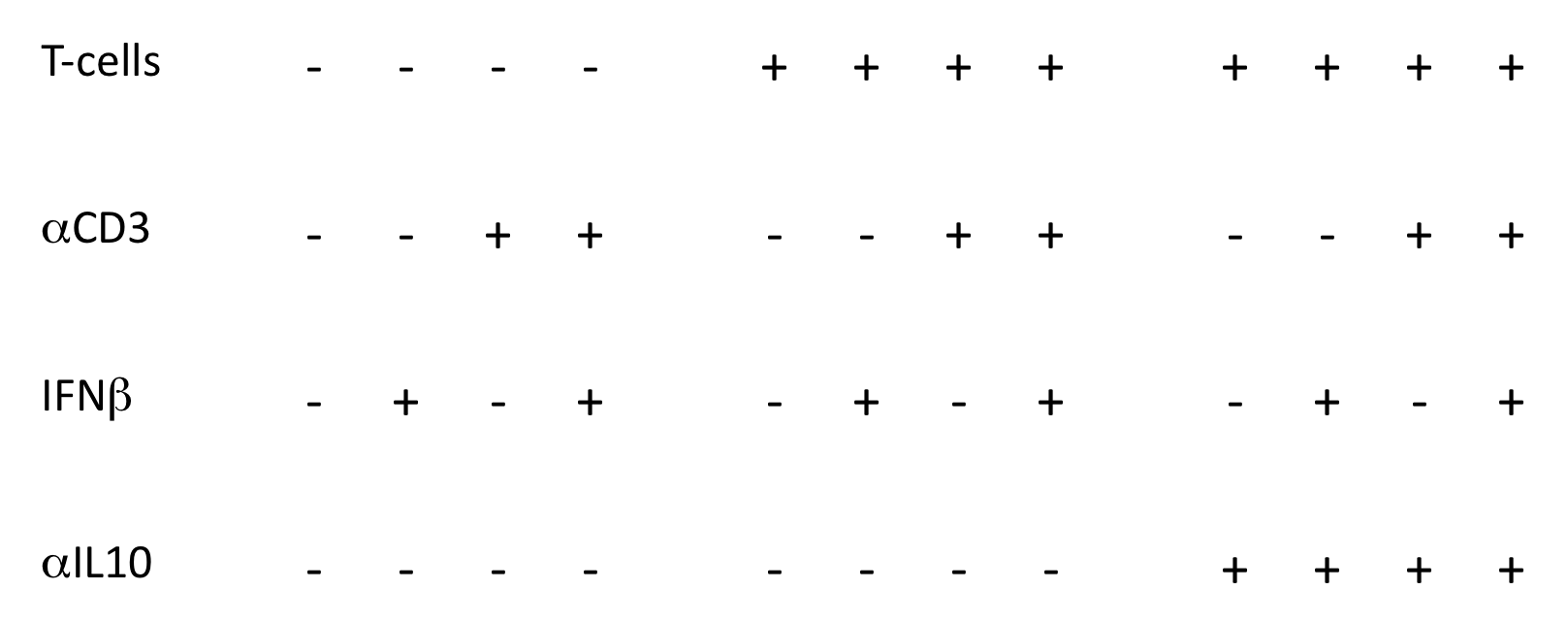


**Fig.S5** mRNA expression of *IL1B* is inhibited by co-incubation of monocytes with T-cells in the presence of αCD3 and IFNβ, this inhibition is reversed by addition of an IL-10 blocking antibody (n=3; *p<0.05 employing repeated measures ANOVA with post-hoc Bonferroni adjustment for multiple comparisons to avoid random correlations).
