# Supplementary material for: Activated Human CD4+CD45RO+ Memory T-Cells Indirectly Inhibit NLRP3 Inflammasome Activation through Downregulation of P2X7R Signalling
Source: PLoS One. 2012 Jun 29;7(6):e39576. doi: 10.1371/journal.pone.0039576 (PMC3387029; doi:10.1371/journal.pone.0039576)
Supplement: Figure S6 — No differences in extracellular LDH levels were detected in the co-cultures of monocytes with CD4+CD45RO+ memory T-cells (n = 3). (DOCX) [file pone.0039576.s006.docx]

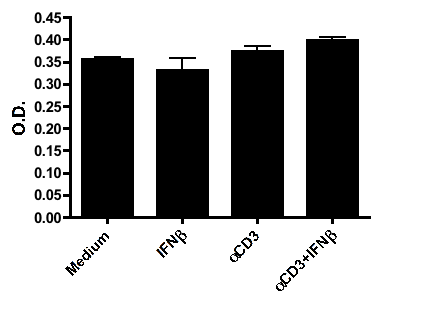


**Fig.S6** No differences in extracellular LDH levels were detected in the co-cultures of monocytes with CD4+CD45RO+ memory T-cells (n=3).
